# Traditional Bullying, Cyberbullying and Mental Health in Early Adolescents: Forgiveness as a Protective Factor of Peer Victimisation

**DOI:** 10.3390/ijerph15112389

**Published:** 2018-10-28

**Authors:** Cirenia Quintana-Orts, Lourdes Rey

**Affiliations:** Faculty of Psychology, University of Málaga, 29071 Málaga, Spain; lrey@uma.es

**Keywords:** bullying, cyberbullying, forgiveness, suicidality, life satisfaction, early adolescence

## Abstract

Traditional and online bullying are prevalent throughout adolescence. Given their negative consequences, it is necessary to seek protective factors to reduce or even prevent their detrimental effects in the mental health of adolescents before they become chronic. Previous studies have demonstrated the protective role of forgiveness in mental health after several transgressions. This study assessed whether forgiveness moderated the effects of bullying victimisation and cybervictimisation on mental health in a sample of 1044 early adolescents (527 females; *M* = 13.09 years; *SD* = 0.77). Participants completed a questionnaire battery that measures both forms of bullying victimisation, suicidal thoughts and behaviours, satisfaction with life, and forgiveness. Consistent with a growing body of research, results reveal that forgiveness is a protective factor against the detrimental effects of both forms of bullying. Among more victimised and cybervictimised adolescents, those with high levels of forgiveness were found to report significantly higher levels of satisfaction compared to those with low levels of forgiveness. Likewise, those reporting traditional victimisation and higher levels of forgiveness levels showed lower levels of suicidal risk. Our findings contribute to an emerging relationship between forgiveness after bullying and indicators of mental health, providing new areas for research and intervention.

## 1. Introduction

Bullying is generally defined as repeated acts of face-to-face aggression in which one or more people intentionally cause pain or harm through a dominance-submission relationship and through the law of silence [1,2]. In recent years, digital advances have made possible negative social interactions in cyberspace, as opposed to bullying in the school setting, known as cyberbullying [3,4].

Both forms of aggression are considered a global public health issue [5] concerning the negative effects on an adolescent’s psychological development, which often persist into adulthood [6,7]. It is well established that students who are bullied or cyberbullied by their peers have a higher risk of depression, suicidal thoughts and behaviours, loneliness, lower levels of self-esteem and less life satisfaction [8,9].

### 1.1. Bullying, Suicide Risk and Life Satisfaction

Bullying and cyberbullying victimisation have long been associated with an increased risk of both suicidal ideation and suicide attempts in cross-sectional and longitudinal studies [9,10]. In light of these studies, research found that the victims of bullying and cyberbullying are at a higher risk of suicidal ideation and suicide attempts than the non-victimised counterparts [11,12], even for both early and older adolescents, as well as for boys and girls [9]. The majority of research on suicide has focused on the identification of factors that increase the risk of this behaviour [13]. In contrast, we know very little about the factors that decrease the likelihood of suicidal behaviour among those exposed to known risk factors such as bullying victimisation [14].

Based on the definition of mental health being more than the absence of health and psychological problems [15], other constructs have been linked to the presence of positive outcomes such as life satisfaction; these are defined as the subjective appraisal of life taken as a whole [16]. The extant literature reveals that bullying victimisation is associated with poorer life satisfaction among adolescents [17,18]; for instance, adolescents who experienced both traditional bullying and cyberbullying [19] experience lower life satisfaction ratings in comparison with adolescents who were not victimised. Indeed, Moore et al. [17] found that students who reported being cyberbullied also indicated having lower life satisfaction with school, environment, and family compared to those who were not cybervictimised. 

Several areas of research have been seek to explain how and why bullying might decrease mental health among victims. For example, some researchers have examined the emotions associated with experiences of being bullied, identifying loneliness, sadness, embarrassment, being stressed or depressed as factors of bullying victimisation [20,21]. Specifically, some scholars have investigated the emotional impact that both traditional and cyberbullying has on victims, reporting that negative emotions were less frequently reported for cyberbullying, in comparison with traditional bullying [20]. Drawing on the General Strain Theory (GST), Hay and Meldrum [22] argue that victimisation is a type of strain that might be related to more negative emotions, as well as harmful behaviours directed at oneself. Consequently, deficiency in negative emotion regulation and/or inappropriate emotional expression styles could be risk factors for victims of bullying to poor mental health. Thus, it is argued that an effective emotional response by victims can also play a part in the success of intervention programs [20,23]. 

Because bullying and cyberbullying victimisation are known mental health risk factors among adolescents [8,14], it is important to consider the vulnerability of this age group and any protective factors that might be associated with psychological adjustment. In this sense, protective factors lessen the likelihood of bullying victimisation and mediate or moderate the effects of risk factors [4]. Recent attention on the negative outcomes of both traditional and online victimisation have prompted some researchers to examine protective factors that could potentially be the focus of an intervention [23,24]. Although different protective factors (e.g., social support, academic achievement or coping strategies) have been proposed to influence the association between victimisation and diverse forms of health difficulties [24,25,26], there are still numerous knowledge gaps regarding factors that could potentially protect adolescents exposed to bullying victimisation [27]; as a consequence, the previous literature has tended to focus on risk, rather than protective, factors [24,28]. Thus, it is important to pursue endeavors that determine protective personal factors as appropriate points of intervention in order to mitigate the impact of negative emotions on mental health associated with bullying and cyberbullying victimisation. One individual approach to dealing with negative emotions after interpersonal transgressions that has been found to have related to less emotional hurt in response to past different instances of aggression is forgiveness [29,30]. Although there is a growing body of research focusing on forgiveness and bullying, the majority of studies have been conducted in the context of adulthood [31,32]. Consequently, this study focuses on forgiveness as one potentially important individual protective factor to effectively contribute to the scarce existing literature.

### 1.2. Forgiveness as a Protective Factor

Forgiveness is a concept that involves: “(a) the reduction in vengeful and angry thoughts, feelings, and motives that may be accompanied by (b) an increase in some form of positive thoughts, feelings, and motives” ([33], p. 154) towards the perpetrator, the transgression, and oneself [29]. Previous studies have suggested the beneficial effects of forgiveness in psychological and physical health in a variety of interpersonal offenses, most of which are focused on adult forgiveness [29,31]. 

During the last decade, there has been a growing interest in the association between forgiveness and bullying [30,34]. According to a recent systematic review by Quintana-Orts, Rey, and Worthington [32], forgiveness was negatively related with bullying victimisation and predicted better psychological adjustment, even after accounting for bullying behaviour, victimisation experiences, and general coping strategies (e.g., conflict resolution, advice, and seeking support). Furthermore, forgiveness was also found to be a moderator, showing that it might reduce the impact of the negative consequences of being bullied. For example, Liu et al. [35], who focused on the association between bullying victimisation and suicidal ideation, found that victimised adolescents with greater levels of forgiveness reported lower levels of suicidal ideation compared to those with low levels of forgiveness. From the stress-and-coping framework, forgiveness is argued to be an effective emotion-focused coping strategy [36] at reducing the sense of threat appraisal and the level of negative emotions and motivations related to bullying victimisation [26,30]. In this sense, forgiveness might be an emotion-focused coping strategy that would allow adolescents to overcome resultant emotional hurt, decreasing health and psychological problems linked to be bullied and increasing psychological adjustment and wellbeing [32,37]. 

Regardless of life satisfaction and suicide risk as relevant indicators of the mental impact of traditional and online bullying in adolescents [5], few studies have individually examined the potential mitigating factors of the effects of traditional and online victimisation. From the positive psychology framework, it is particularly important to consider the individual factors that could act as a buffer for bullied adolescents; instead of simply protecting adolescents from experiencing bullying, these factors could help them to understand how individual factors provide protection against the deleterious consequences of interpersonal harm [28]. In addition, studies that explore the importance of forgiveness associated with victimisation are scarce and based on findings from adult population literature. Thus, the current study aimed to add to this body of evidence regarding how forgiveness is related to improved mental health after bullying transgressions. To our knowledge, no studies have specifically measured how forgiveness levels in adolescents can mitigate the consequences of victimisation when it comes to bullying and cyberbullying.

### 1.3. Current Study

The aim of this study was explorative in order to extend the previous research regarding the relationship between bullying victimisation, forgiveness, and mental health in adolescents, given the scarcity of studies. Given that forgiveness is a positive concept associated with psychological adjustment in adolescents [38,39], and consistent with the notion that forgiveness might also buffer or weaken the association between victimisation and mental health [35], our hypotheses were the following:

(1) We expected to find evidence for the unique role of forgiveness in predicting both suicide risk and life satisfaction, even after accounting for variance attributed to the co-existence of victimisation [40,41].
(2) We expected that forgiveness would moderate the relationship between both forms of bullying victimisation and mental health (i.e., suicide risk and life satisfaction).

## 2. Materials and Method

### 2.1. Participants

The reference population used to conduct the current study comprised adolescents aged between 12 and 14 (*N* = 190,773) in Andalusia (an autonomous community located in southern Spain). The sample sized was estimated at 1040 students, with a sampling error of ±3.03%, a confidence level of 95%, and *p* = 0.5. 

The current study consisted of 1044 early adolescents (517 males and 527 females) from six state-run secondary schools in Malaga (south of Andalusia, Spain). The sample was predominantly Spanish (86.2%). The age of participants ranged from 12 to 14 years, with a mean age of 13.09 years (*SD* = 0.77). Of these adolescents, 82.4% were enrolled in the first cycle of Compulsory Secondary Education (CSE).

### 2.2. Measures

#### 2.2.1. Independent Variables

Traditional bullying victimisation. The European Bullying Intervention Project Questionnaire (EBIPQ) [42], a 7-item instrument providing a measure of traditional peer victimisation using a 5-point Likert scale to represent the frequency of bullying over the previous two months (ranging from 0 = never to 4 = more than once a week). This measure includes the instruction “In this section we ask you about your possible experiences related to bullying in your environment (school centre, friends, acquaintances), as a victim and/or aggressor. Your answers will be confidential” and the base question “Have you participated in the following behaviour in the last 2 months?”. Items included physical and verbal behaviours, psychological abuse and social exclusion (e.g., “someone has hit, kicked, or pushed me”). The EBIPQ has demonstrated good psychometric properties [42]. The Cronbach’s alpha was 0.83 in this study.

Cyberbullying victimisation. The European Cyberbullying Intervention Project Questionnaire ECIPQ [42] comprised 11 items and covered cybervictimisation over the previous two months, with the same five response options as in the previous questionnaire (ranging from 0 = never to 4 = more than once a week). This measure includes the instruction “In this section we ask you about your possible experiences related to cyberbullying in your environment (school centre, friends, acquaintances), as a victim and/or aggressor. Your answers will be confidential” and the base question “Have you participated in the following behaviour either online or through mobile phones in the last 2 months?”. Items included online harassment, the distribution and/or alteration of embarrassing images or videos, and indirect abuse (e.g., “someone has said nasty things to me or insulted me via e-mail or SMS”). The Spanish version has shown strong reliability and validity [42]. The Cronbach’s alpha value of this measure was 0.85 in this study.

#### 2.2.2. Dependent Variables

Suicidal Risk. Suicidal risk was measured using the SBQ-R [43]. The SBQ-R is a 4-item scale that provides an indication of overall suicidality on a Likert scale assessing lifetime suicide ideation and attempts, the frequency of suicide ideation in the past year, communication of suicidal intent, and the likelihood of future suicidal behaviour. The response options vary per item. For instance, respondents are asked to indicate their frequency of having suicidal ideations, ranging from 1 = never to 5 = very often. The SBQ-R was translated from English into Spanish using the back translation method, which has previously demonstrated good reliability and validity in Spanish samples [44]. The Cronbach’s alpha was 0.87 in this study.

Life satisfaction. The SWLS [45] is a widely used assessment of overall life satisfaction comprising five self-referencing statements, with responses represented on a 7-point Likert scale. An example of the items included is “In most ways my life is close to my ideal”. In the present study, a well-validated Spanish version of the SWLS was used [46]. The Cronbach’s alpha value of this measure was 0.84.

#### 2.2.3. Moderator

Forgiveness. The Values in Action Inventory for Youth (VIA-Youth) [47], a 10-item forgiveness questionnaire, provided a measure of forgiveness using a 5-point Likert scale ranging from 1 (not at all) to 5 (completely). An example of an item is: “I always let bygones be bygones”. The Spanish version of the subscale was used, which can be found on the Authentic Happiness website (https://www.authentichappiness.sas.upenn.edu/testcenter). This scale has demonstrated good psychometrics properties in different studies [48]. The Cronbach’s alpha was 0.72 in this study.

#### 2.2.4. Control Variables

In addition to the aforementioned measures, the analyses also included demographic items (sex and age) to control for a number of potentially important spurious relationships.

### 2.3. Procedure

A convenience sampling method was used to collect data during a single period from February to May 2017. Consent was obtained from participants’ families, and anonymity and confidentiality of the information was assured. Participants received a paper-and-pencil questionnaire which was administered in a 30-min session to the classes as a whole. Students were also informed of the general purpose of the study and of its confidential, voluntary, and anonymous nature. Indeed, the anonymity and confidentiality were ensured by not collecting any personally identifiable information such as name, email, home. One researcher and at least one teacher from the school were present while students completed all questionnaires. Fifty-eight questionnaires were excluded due to missing data or unreliable answers (e.g., one-sided response patterns, such as consistently filling in the extremes or with many measures unanswered). This procedure was carried out in accordance with Declaration of Helsinki (2013) and approved by the Ethics Committee at the University of Málaga (CEUMA; approval number 62/2016-H).

### 2.4. Data Analyses 

**Figure 1:**
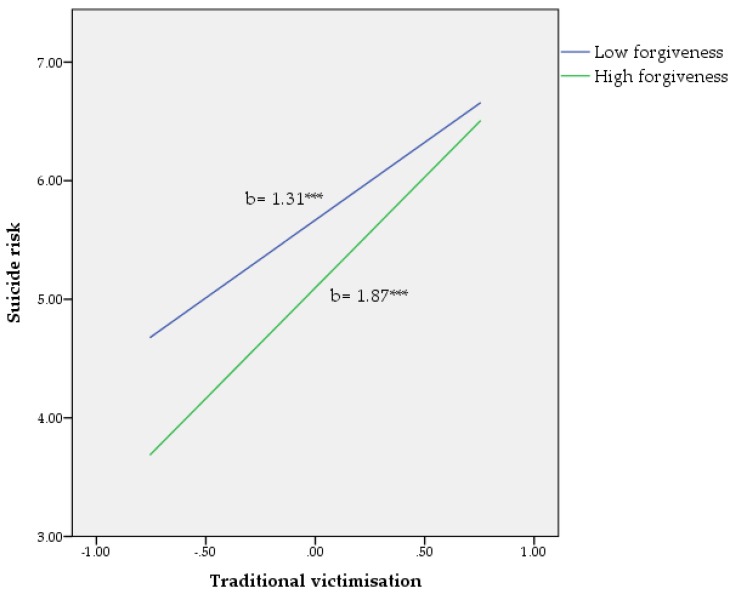
Relationship of traditional victimisation and forgiveness for predicting suicide risk. *** *p* < 0.001. Note: values for forgiveness (moderator) are the mean and plus/minus one standard deviation (*SD*) from the mean.

**Figure 2:**
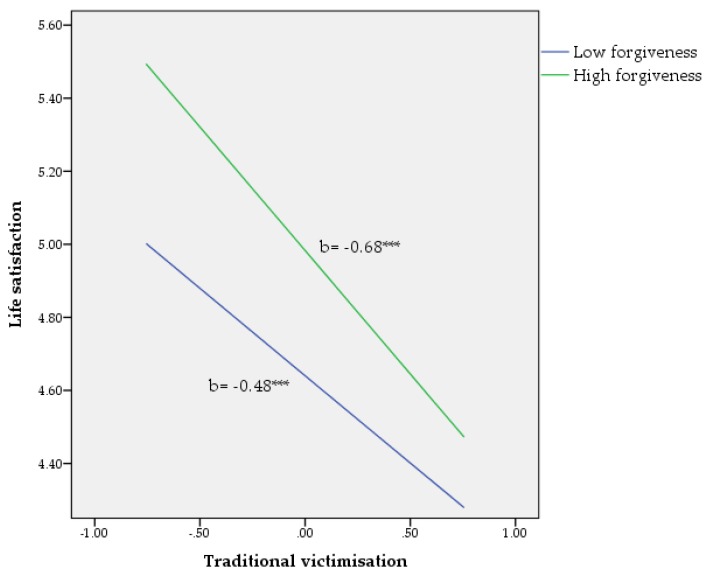
Relationship of traditional victimisation and forgiveness for predicting life satisfaction. *** *p* < 0.001. Note: values for forgiveness (moderator) are the mean and plus/minus one standard deviation (*SD*) from the mean.

**Figure 3:**
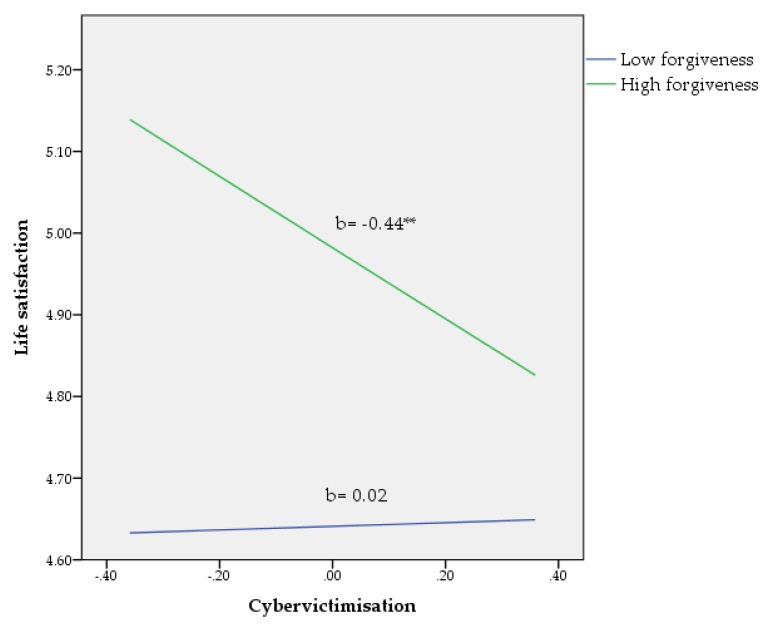
Relationship of cybervictimisation and forgiveness for predicting life satisfaction. ** *p* < 0.01. Note: values for forgiveness (moderator) are the mean and plus/minus one standard deviation (*SD*) from the mean.

Data were analysed using SPSS version 22. Descriptive statistics following Pearson’s correlation analyses were conducted to establish significant associations among forgiveness, traditional victimisation, cybervictimisation, depression, and suicidal thoughts and behaviours (see Table 1). Subsequently, to address the moderation effects between both traditional and cybervictimisation and forgiveness for predicting suicidal thoughts and behaviours and life satisfaction, SPSS’s PROCESS macro was used [49] (Table 2 and Table 3). First, we examined the effect of the control variables (age and sex) and the concurrent form of bullying experience on traditional bullying and cyberbullying, respectively. Finally, the effect size of the interaction term (*f* ^2^) was calculated to determine the unique variance [50], and the figures were plotted to visually inspect the moderating effects (see Figure 1, Figure 2 and Figure 3) [49]. According to Hayes [49], the values for forgiveness, as quantitative moderator, are the mean and plus/minus one standard deviation (*SD*) from mean. 

## 3. Results

### 3.1. Descriptive Analysis

Before performing the moderating analyses, bivariate correlations among study measures were computed (Table 1). Consistent with previous findings, traditional victimisation and cybervictimisation were positively and significantly related to suicide risk and negatively associated with life satisfaction. Moreover, forgiveness was negatively and significantly related to suicide risk and positively associated with life satisfaction. 

Regarding the sex difference analyses, independent t-tests alone revealed higher scores in cybervictimisation (t(1044) = −2.48, *p* < 0.05, d = −0.17), forgiveness (t(1044) = −2.53, *p* < 0.05, d = −0.16), and suicide risk (t(1044 )= −6.04, *p* < 0.001, d = −0.37) for girls compared to boys. According to Cohen’s magnitude difference criteria, a cut-off point of 0.2 is considered to be a “small” minimum effect size, whereas 0.5 is the minimum “medium” effect size, and 0.8 is the minimum “large” effect size. In our study, therefore, the effect size of the sex differences in forgiveness and cybervictimisation were considered small, whereas in suicide risk the effect size was between small and moderate.

### 3.2. Moderation Test

Finally, to examine whether traditional victimisation and cybervictimisation have different effects on suicide risk and life satisfaction, depending on their level of forgiveness, four ordinary least squares (OLS) regression analyses were estimated using SPSS’s macro PROCESS ([49]; 5000 bootstrapped samples). As the sex differences in forgiveness (moderator) were considered “small”, moderator analyses were made for the whole sample. Sex, along with age and the remaining form of bullying victimisation, was entered as a covariate. Additionally, effect size statistics (*f*^2^) were calculated to determine the unique variance explained by the interaction term. The effect size results obtained for moderator effects can be interpreted as small (*f* ^2^ = 0.005), medium (*f* ^2^ = 0.01), and large effects (*f* ^2^ = 0.025) [51]. Lastly, to test for the potential moderating effects of forgiveness, we used the procedure described by Hayes and Matthes [52] to visually inspect the significant interactions. 

#### 3.2.1. Traditional Victimisation

Suicidal risk. As seen in Table 2, the full model, including the covariates (sex, age, and cybervictimisation), the main variables, and the interaction term, accounted for 48% of the variance in suicidal thoughts and behaviours (F(6, 1033) = 50.37, *p* < 0.001). Within the variable set, sex (*p* < 0.001), cybervictimisation (*p* < 0.001), traditional victimisation, (*p* < 0.001) and forgiveness (*p* < 0.01) were potentially significant predictors of suicide risk. Finally, a significant interaction effect between traditional victimisation and forgiveness (b = −0.36, *p* < 0.05) was found to be significant for a small (*f*^2^ = 0.004) amount of additional variance in suicide risk after controlling for covariates, traditional victimisation, and forgiveness (see Figure 1).

Life satisfaction. As seen in Table 2, the total model, including the covariates, the main variables, and their interactions, accounted for 39% of the variance in life satisfaction (F(6, 1037) = 31.16, *p* < 0.001). In short, no covariate effects were found for sex (*p* = 0.09), age (*p* = 0.13), or cybervictimisation (*p* = 0.19). Nonetheless, there were significant effects found for both traditional victimisation (*p* < 0.001) and forgiveness (*p* < 0.001). Finally, there was a significant interaction effect between traditional victimisation and forgiveness in explaining unique variance in life satisfaction (b= −0.13, *p* < 0.05). This interaction accounted for a small (*f*^2^ = 0.003) percentage of additional variance in life satisfaction after controlling for covariates and both potential predictors (see Figure 2).

#### 3.2.2. Cybervictimisation

Suicidal risk. Table 3 demonstrates that the total model, including the covariates (sex, age, and cybervictimisation), the main variables, and the interaction term, accounted for 47% of the variance in suicide risk (F(6, 1033) = 50.00, *p* < 0.001). In short, sex (*p* < 0.001), traditional victimisation (*p* < 0.001), cybervictimisation (*p* < 0.001), and forgiveness (*p* < 0.01) were found to be potentially significant predictors of suicide risk. Nonetheless, no significant interaction effect was found between cybervictimisation and forgiveness (*p* < 0.08) for explaining unique variance in suicidal risk. 

Life satisfaction. As can be seen in Table 3, the total model, including the covariates, the main variables, and their interactions, accounted for 39% of the variance in life satisfaction (F(6, 1037) = 31.52, *p* < 0.001). Within the variable set, just traditional victimisation (*p* < 0.001) and forgiveness (*p* < 0.001) were found to be potentially significant predictors of life satisfaction. Finally, a significant interaction between cybervictimisation and forgiveness (b= −0.30, *p* < 0.05) accounted for a small (*f*^2^ = 0.005) amount of variance in life satisfaction after controlling for covariates, cybervictimisation, and forgiveness (see Figure 3).

## 4. Discussion

In light of the many studies that have aimed to evaluate the relationship between forgiveness and specific mental health variables, the aim of this study was to analyse the association between bullying victimisation and forgiveness among early adolescents, whilst also taking into account concurrent forms of victimisation and sociodemographic variables (i.e., age and sex). We studied victimisation of both traditional bullying and of cyberbullying; further, we analysed the moderator role of forgiveness in the relationship between both forms of victimisation and mental health to clarify its protective role. 

Consistent with previous findings, associations between forgiveness and mental health were found among adolescents [38,39,53]; forgiveness was negatively and significantly related to suicide risk and positively associated with life satisfaction. Further, and more importantly, results revealed that students with higher levels of forgiveness were less likely to report mental health problems after experiencing bullying (both in its traditional and online forms) compared to those adolescents with low levels of forgiveness.

That bullying victimisation would be associated with detrimental mental health is neither a new, nor an unexpected, finding [5,40]. However, the fact that willingness to forgive others, the situation, and oneself is associated with a lower suicide risk and with higher levels of life satisfaction is significant. According to Worthington and Scherer [36], forgiveness provides a useful emotion-focused coping solution to combat the stressful and negative emotional effects of detrimental emotions and motivations. Our findings provide support for our first hypothesis confirming that forgiveness is related to better mental health, not only for those who are frequently victimised but for those who experience any victimisation at all; this could help the victims of bullying overcome any emotional damage it caused [30,37]. Thus, these results help us to understand how positively managing negative emotions and post-bullying behaviour is related to the presence of forgiveness and appears to provide a buffer against the detrimental effects of bullying on mental health, as it might offer another perspective for bullying intervention [37]. 

However, whilst in traditional bullying victimised adolescents with high levels of forgiveness report less suicide risk and higher life satisfaction; in cyberbullying, forgiveness alone seems to be an important protective factor against negative impacts on life satisfaction. Thus, our second hypothesis was partially supported. One plausible explanation is related to the type and severity of the offense [54,55]. Victims of direct forms of traditional bullying scored highly on negative emotions (such as anger, embarrassment, and stress, etc); whereas victims of cyberbullying reported negative emotions less frequently [56]. Along this line, victims of directly aggressive offenses report fewer instances of forgiveness-related motivations than victims of less directly aggressive transgressions [55]. Thus, one possibility is that traditional bullying is perceived as a more damaging form of bullying than cyberbullying [56], affecting the perception of the seriousness of the offense and its reactions. It would be of interest for future studies to investigate this finding further. 

In summary, we examined and found support for the idea of forgiveness as a buffer against the detrimental health consequences of bullying in adolescents. Consistent with past research [38,53], we found that protective factors (i.e., high levels of forgiveness) were consistently associated with the lowest risk of psychological maladjustment. Together, our findings not only underscore a need for researchers to consider the important role of positive individual factors as protective variables in a bullying context, but also highlight the potentially greater costs associated with the absence of protective factors over the potential benefits associated with the presence of them in adolescents at risk of traditional and cybervictimisation.

### 4.1. Limitations

Although the present study represents an advance in the field of forgiveness in mental health among bullied adolescents, some limitations require consideration. First, our research was cross-sectional, making it impossible to make causal inferences or conclusions about the relationships among study variables. Future research may identify forgiveness as a protective factor in experiences of bullying through longitudinal research; for example, how forgiveness influences the relationship between victimisation and mental health after a bullying or cyberbullying experience, and the impact of intervention programmes. Furthermore, it is also important to note the inherent limitations of asking adolescents to self-report their behaviours due to the implied bias of social desirability. Another limitation is that data was collected from a convenience sample, so generalization to the adolescent population may be biased. Although it is a large sample from six high schools, permission from parents to enable adolescents to take part may result in a non-random sample of students. Finally, it would be interesting to examine other variables such as how the severity of the bullying experience(s) [55] influences the relationship between forgiveness and mental health. Future research should continue to identify specific causal paths between forgiveness, bullying, and mental health. 

### 4.2. Implications of Findings on Research and Practice

Given the role of bullying victimisation and forgiveness in the onset of mental health, understanding the predictive role of forgiveness in the impact of victimisation in a general population of early adolescents could provide important information about mental health factors and prevention strategies. This result, together with the different moderating role of socio-emotional personal factors (e.g., self-management and emotional intelligence) between types of bullying victimisation, has several relevant implications for researchers and educators when developing effective bullying interventions [40]. 

Although current meta-analyses and systematic reviews have reported the effectiveness of anti-bullying programs, there are several adolescents that continue to be victimized for a prolonged period even after an involvement in a universal school-based intervention [21]. To prevent the persistency, our results suggest that forgiveness could potentially be used as an important adjunct to current approaches for increasing mental health in bullied adolescents. Our findings highlight that adolescents have positive individual characteristics that may be developed to promote the recovery of bullied adolescents. As some authors have already emphasized [30,37], it would be useful to include evidence-based interventions on forgiveness in the field of anti-bullying interventions to increase empathy among adolescents and empowerment among victims. In addition, interventions may also benefit from strategies to decrease negative feelings and aggressive reactions (e.g., motivations to revenge) for particularly vulnerable adolescents [26] by improving more positive thoughts, behaviours and feelings with their peers and including tailored strategies to better stimulate social interactions even after an offense or an interpersonal conflict [55]. Future research in this field should clarify core concepts underpinning the intervention. For instance, emphasizing to adolescents that forgiveness does not necessarily involve reconciliation, tolerating, condoning, or excusing aggressive behaviour. This understanding of forgiveness would serve to protect victims of aggression from further unhealthy relationships, even when aggressions continues in schools, boosting courage to report the bullying aggressions. Finally, interventions may benefit from parental and teacher components to broaden the scope of the intervention, such as actively involving parents and teachers to improve forgiveness in adolescents instead of calling for revenge or avoidance [32,57].

## 5. Conclusions

The current findings constitute a step forward in understanding forgiveness as a personal protective factor of bullied adolescents. Although previous bullying experience, which is an unchangeable variable, could be responsible for a decrease in the mental health of adolescents, this counterproductive effect could be mitigated by levels of forgiveness. Thus, forgiveness, together with other protective factors (e.g., self-esteem and emotional intelligence), should be taken into account in the design of targeted interventions. Along this line, our findings might be helpful for advancing knowledge in the field of potential protective factors and in making intervention recommendations in incidences of bullying victimisation during adolescence. Efforts should continue to identify protective factors and help victims of bullying, as well as to create effective bullying prevention and intervention programmes.

## Figures and Tables

**Table 1:** Means, *SD*s and intercorrelations among measures.

	Mean (*SD*)	1	2	3	4	5
1. Traditional victimisation	0.80 (0.76)	-				
2. Cybervictimisation	0.18 (0.36)	0.60 ***	-			
3. Forgiveness	3.41 (0.77)	−0.04	-0.04	-		
4. Suicidal thoughts and behaviours	5.37 (3.73)	0.42 ***	0.35 ***	−0.08 **	-	
5. Life satisfaction	4.32 (3.84)	−0.36 ***	−0.26 ***	0.14 ***	−0.49 ***	-

** *p* < 0.01, *** *p* < 0.001.

**Table 2:** Tested moderation models with suicidal risk and life satisfaction as outcomes predicted by traditional victimisation, forgiveness and multiplicative interaction terms.

	B	SE	R^2^	Δ R^2^	95% CI
**Model 1. Suicidal risk**			0.48 ***		
Constant	0.78	1.81			−2.76 to 4.32
Sex	1.29 ***	0.21			0.89 to 1.69
Age	0.18	0.14			−0.08 to 0.45
Cybervictimisation	1.38 ***	0.36			0.67 to 2.08
Traditional victimisation	1.59 ***	0.17			1.26 to 1.92
Forgiveness	−0.37 **	0.14			−0.64 to −0.11
Traditional victimisation × forgiveness	−0.36 *	0.16		0.004 *	0.04 to 0.68
**Model 2. Life satisfaction**			0.39 ***		
Constant	6.08 ***	0.70			4.72 to 7.44
Sex	−0.13	0.08			−0.29 to 0.02
Age	−0.08	0.05			−0.18 to 0.02
Cybervictimisation	−0.18	0.14			−0.45 to 0.09
Traditional victimisation	−5.78 ***	0.07			−0.71 to −0.45
Forgiveness	0.22 ***	0.05			−37.55 to −23.57
Traditional victimisation × forgiveness	−0.13 *	0.06		0.003 *	−0.25 to −0.01

* *p* < 0.05, ** *p* < 0.01, *** *p* < 0.001. Note: b = Unstandardized beta; SE = Standard error of beta coefficients.

**Table 3:** Tested moderation models with suicidal risk and life satisfaction as outcomes predicted by cybervictimisation, forgiveness and multiplicative interaction terms.

	B	SE	R^2^	Δ R^2^	95% CI
**Model 1. Suicidal risk**			0.47 ***		
Constant	−0.29 ***	1.82			−3.86 to 3.28
Sex	1.27 ***	0.21			0.87 to 1.68
Age	0.19	0.14			−0.08 to 0.46
Traditional victimisation	1.57 ***	0.17			1.23 to 1.90
Cybervictimisation	1.44 ***	0.36			0.74 to 2.15
Forgiveness	−0.36 **	0.14			−0.63 to −0.10
Cybervictimisation × forgiveness	0.56	0.32		0.002	−0.06 to 1.18
**Model 2. Life satisfaction**			0.39 ***		
Constant	6.54 ***	0.70			5.17 to 7.91
Sex	−0.13	0.08			−0.28 to 0.03
Age	−0.08	0.05			−0.69 to −0.44
Traditional victimisation	−0.57 ***	0.07			−0.01 to 0.22
Cybervictimisation	−.21	0.14			−0.48 to 0.07
Forgiveness	0.22 ***	0.05			0.12 to 0.33
Cybervictimisation × forgiveness	−0.30 *	0.12		0.005 *	−0.54 to −0.06

* *p* < 0.05, ** *p* < 0.01, *** *p* < 0.001. Note: b = Unstandardized beta; SE = Standard error of beta coefficients.
